# Protein interaction studies of curli fimbriae in Escherichia coli biofilms

**DOI:** 10.6026/97320630015918

**Published:** 2019-12-31

**Authors:** Maithreyi Suresh Iyer, PA Abhinand, CR Hemalatha

**Affiliations:** 1 Department of Bioinformatics, Sri Ramachandra Institute of Higher Education and Research, Porur, Chennai-600 116, India

**Keywords:** CAUTIs, Escherichia Coli, Biofilms, Curli, Protein-Protein Interaction, Protein Modeling, Drug Discovery, Combinatorial Library

## Abstract

Catheter-associated urinary tract infections (CAUTIs) caused by biofilms on indwelling medical devices are the most common type of nosocomial infections, a major health concern due to complications and frequent recurrence. The infections are most often caused by Escherichia coli. Curli are proteinaceous components of a complex extracellular matrix produced by various strains of Enterobacteriaceae. Curli fibers are involved with adhesion to surfaces, cell aggregation and biofilm formation. Therefore, it is of interest to study the protein interactions in curli biogenesis, identifying proteins involved in curli biogenesis, the interactions and development of a combinatorial library of novel lead molecules against biofilm formation by Escherichia coli. Targeting the CsgG protein of Escherichia coli could provide new treatment modalities to fight CAUTIs, better. This study may also help study infections caused by various strains of Enterobacteriaceae, in general.

## Background

A biofilm is made up of a polysaccharide matrix embedded with microbial cells that associate with a surface irreversibly [1,2]. Cells growing in biofilms are found to be more resistant to biocides, antibiotics, desiccation and oxidative stress [3]. Biofilms are often found on medical devices and this is a major concern for medical professionals [4]. Three fourths of UTIs acquired in a hospital are usually associated with urinary catheters. Approximately 15 - 25% of internal patients have catheters in place during their hospital stay [5]. One of the most common type of nosocomial infections are caused by CAUTIs due to complications and the capacity to recur. Escherichia coli causes most of these infections. [6]. Curli is the complex of proteins formed in the extra-cellular matrix produced by many organisms of Enterobacteriaceae. These curli proteins are responsible for surface adhesion, aggregation of cells and formation of biofilms [7]. Therefore, it is of interest to study curli biogenesis and the protein-protein interactions involved in the biofilm formation. We further use a combinatorial library of lead molecules derived from existing literature of phytochemicals to describe hits with proven activity against curli formation in E. coli biofilms [8].

## Methodology

### Protein-protein interactions:

The protein-protein interactions were obtained for each target protein individually. The interactions were studied and the common genes were grouped together. The results were tabulated on excel sheets.

### Homology modeling of csgg:

The FASTA sequence of the carrier protein csgG was obtained from PDB. CsgG protein was modeled using Swiss-Model in the alignment mode. The template chosen had 100% sequence identity and 87% query coverage. The model obtained was analyzed using RAMPAGE, ERRAT and ProSA.

### Evaluation of modelled protein:

From a comprehensive literature survey, four compounds that target curli biogenesis were identified. A common scaffold was obtained using Marvin Sketch and this scaffold was given as the input for production of a combinatorial library using SmiLib. The 60 compounds obtained were further analyzed for their chemical properties using ChemSketch.

## Results and Discussion:

Among UTIs acquired in the hospital, approximately 80% were estimated to be associated with urinary catheters [9]. Catheter-associated urinary tract infections (CAUTIs) are a major health concern due to the complications and frequent recurrence. Several studies have shown the association of biofilms in establishment of CAUTIs. E. coli was found to be the most predominant organism in causing UTIs. Using this knowledge, we further studied E. coli biofilms and identified a component essential in formation of biofilms; Curli fimbriae.

## Target identification - studying protein-protein interactions involved in etiology of curli formation:

**Figure 1:**
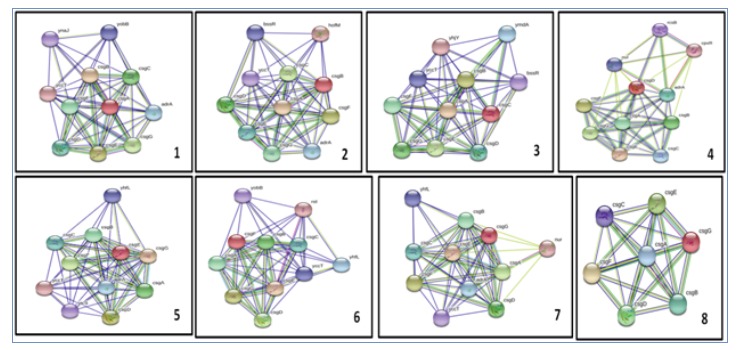
Individual and combined protein interaction networks of csgA, csg-B, csg-C, csg-D, csg-E, csg-F and csg-G obtained from STRING 11.0

The major proteins involved were studied using STRING database. The common genes were identified. yccT was the most common gene found interacting with six of seven proteins : CsgA, CsgB, CsgC, CsgE, CsgF and CsgG (Figure 1). The expression of yccT was found to be activated by biofilm regulator CsgD [10]. The next most common gene was adrA commonly called dgcC, is part of a regulatory network involved in development of curli. It has interactions with proteins: CsgA,CsgB, CsgD, CsgE, and CsgG [7]. The nur gene commonly called Rpos regulates curli synthesis under oxidative stress conditions and was found to interact with proteins CsgD and CsgG. bssR gene is a regulator of biofilm formation in E. coli. It was found to interact with proteins CsgB and CsgC [11]. This study was carried out to showcase the interactions of genes in curli biogenesis. If these genes are targeted, their interactions may be disturbed which in turn could affect biofilm formation [12]. This however, requires wet lab experiments for confirmation. Literature survey showed that CsgA and CsgB proteins were transported via the carrier lipoprotein CsgG [13].

## Homology modeling of target protein and target evaluation:

The CsgG protein was modeled using the Homology modeling method in the alignment mode. Swiss-Model was used for this purpose and the protein modeled was downloaded. Ramachandran plot analysis was done using RAMPAGE. The results obtained showed 97.5 % in favorable region, 2.5% in allowed region and 0% in outlier region. The results from RAMPAGE show that the protein modeled is acceptable. ProSA validates the protein structures based on X-ray analysis, NMR spectroscopy and theoretical calculations. The modeled CsgG protein was found to agree with the XRD, NMR calculations. The modeled protein was of high quality as it showed a quality factor of 99.087 on ERRAT.

## Creation of combinatorial library of novel molecules to target biofilm formation in E. Coli

Small molecules that inhibit curli biogenesis and biofilm formation were identified [14]. A common scaffold was identified among 4 drug molecules and a combinatorial library was obtained using SmiLib. Out of 60 compounds generated, 48 lead molecules followed Lipinski’s rule of 5 (Table 1). These 48 molecules could be potential target molecules to inhibit biofilm formation in E. coli.

## Conclusion

Urinary Catheter infections (CAUTI) are a major concern due to their drug resistance caused by biofilm formation [15]. The proteins involved in curli biogenesis in Escherichia coli biofilms were identified and their interactions were studied. The common genes that interact with these proteins were identified. Targeting these genes could provide new treatment modalities to fight CAUTIs. CsgG protein was modeled using Swiss-Model in the alignment mode and the structure obtained was analyzed. A common scaffold was obtained from drugs that target Curli biogenesis and a combinatorial library of lead molecules was formed. These lead molecules on further analysis could be used as potential drugs to inhibit biofilm formation on urinary catheters by E. coli.

## Figures and Tables

**Table 1:** Combinatorial library obtained from the common scaffold showing 10 out of 60 lead molecules obtained

S.No.	IUPAC Names	Molecular Weight	logP	Hydrogen donors	Hydrogen acceptor
1	(3S)-3-[(S)-hydroxy(methoxy)methyl]-7-[(naphthalen-1-yl)methyl]-2H,3H,5H-[1,3]thiazolo[3,2-a]pyridin-5-one	353.43476	3.31	1	6
2	(3S)-3-[(S)-(2,2-dimethylpropoxy)(hydroxy)methyl]-7-[(naphthalen-1-yl)methyl]-2H,3H,5H-[1,3]thiazolo[3,2-a]pyridin-5one	409.54108	4.93	1	6
3	(3S)-3-[(S)-hydroxy(2-methoxyethoxy)methyl]-7-[(naphthalen-1-yl)methyl]-2H,3H,5H-[1,3]thiazolo[3,2-a]pyridin-5-one	397.48732	3.26	1	8
4	(3S)-3-[(S)-hydroxy[(4-methoxyphenyl)methoxy]methyl]-7-[(naphthalen-1-yl)methyl]-2H,3H,5H-[1,3]thiazolo[3,2-a]pyridin-5-one	459.5567	4.87	1	8
5	(3S)-3-[(S)-hydroxy[2-(piperidin-1-yl)ethoxy]methyl]-7-[(naphthalen-1-yl)methyl]-2H,3H,5H-[1,3]thiazolo[3,2-a]pyridin-5-one	450.593	4.17	1	7
6	(3S)-3-[(S)-hydroxy[2-(piperidin-1-yl)ethoxy]methyl]-7-[(naphthalen-1-yl)methyl]-2H,3H,5H-[1,3]thiazolo[3,2-a]pyridin-5-one	396.43632	4.17	1	7
7	(3S)-3-[(S)-hydroxy(phenoxy)methyl]-7-[(naphthalen-1-yl)methyl]-2H,3H,5H-[1,3]thiazolo[3,2-a]pyridin-5-one	415.50414	5	1	6
8	(3S)-3-[(S)-hydroxy(2,2,2-trifluoroethoxy)methyl]-7-[(naphthalen-1-yl)methyl]-2H,3H,5H-[1,3]thiazolo[3,2-a]pyridin-5-one	421.4327296	4.26	1	6
9	4-methanesulfonylphenyl (3S)-7-[(naphthalen-1-yl)methyl]-5-oxo-2H,3H,5H-[1,3]thiazolo[3,2-a]pyridine-3-carboxylate	493.59452	3.99	0	8
10	[(2S)-2-methyl-4-oxo-1,3-thiazolidin-3-yl]methyl (3S)-7-[(naphthalen-1-yl)methyl]-5-oxo-2H,3H,5H-[1,3]thiazolo[3,2-a]pyridine-3-carboxylate	468.58836	3.24	0	6
